# An uncommon encounter: crossed fused renal ectopia with singular ureter: a case report

**DOI:** 10.1186/s13256-024-04689-8

**Published:** 2024-08-04

**Authors:** Prajwal Dahal, Kapil Dawadi, Ongden Yonjen Tamang, Sabina Parajuli, Natasha Dhakal

**Affiliations:** 1 Consultant Radiologist, Department of Radiology and Imaging, Grande International Hospital, Kathmandu, Nepal; 2 Resident PGY-1 Pathology, Department of Pathology, Bir Hospital, Kathmandu, Nepal; 3 Manmohan Cardiothoracic Vascular and Transplant Center, Institute of Medicine, Tribhuvan University, Kathmandu, Nepal

**Keywords:** Anomaly, Congenital, Ectopia, Renal, Ureter

## Abstract

**Background:**

Crossed fused renal ectopia (CFRE) is a common congenital anomaly where one kidney is positioned abnormally on the opposite side of the midline, often fused with the other kidney. However, single ureter draining crossed fused renal ectopia is a rare occurrence.

**Case report:**

Here, we report a case of crossed fused renal ectopia with a single ureter in a 46-year-old Nepali male who presented with history of lithuria. Computed tomography revealed that the left kidney was situated on the right side and fused with the right kidney. The renal pelvises of both kidneys were fused, and a single ureter, located on the right side, was draining both kidneys into the bladder. The patient was advised to have regular follow-ups.

**Conclusion:**

Crossed fused renal ectopia with a single ureter represents a rare renal anomaly. Asymptomatic patients can typically be managed conservatively. Regular follow-up is recommended to monitor renal function, calculus formation, infections, and malignant changes.

## Background

Crossed fused renal ectopia (CFRE) commonly appears in routine imaging, where both kidneys are situated on one side of the body and are either partially or fully fused [1]. Typically, CFRE is drained by two ureters, each emptying into its respective side. However, CFRE drained by a single ureter is exceedingly rare, with very few reported cases documented in the literature [2]. In the majority of instances, CFRE remains asymptomatic and is often incidentally discovered during imaging studies. However, some cases may manifest with symptoms such as calculi and infection, attributed to the abnormal positioning of the pelvicalyceal system and ureter, leading to urinary stasis [3]. Moreover, CFRE may be associated with various urological and non-urological abnormalities, including vesicoureteral reflux, pelviureteric junction obstruction, undescended testis, tetralogy of Fallot, sacral agenesis, vaginal agenesis, and anal abnormalities [4].

## Case report

A 46-year-old Nepali male presented to the outpatient department of a tertiary care center with a 2-year history of multiple episodes of lithuria. He reported experiencing right flank pain during the episodes of lithuria, describing it as dull and colicky, radiating to the groin region, moderate in intensity, and exacerbated by movement. Additionally, he mentioned experiencing undocumented fever during these episodes but had not sought medical assistance until now. At the time of presentation, the patient was asymptomatic, and physical and systemic examinations yielded normal results. Blood and urine investigations showed no apparent abnormalities. The patient had no other illnesses. He was mentally fine and stable. There was no history of hereditary and non-hereditary illness in the family. He was from a middle class family and could afford the hospital expenses.

**Fig. 1:**
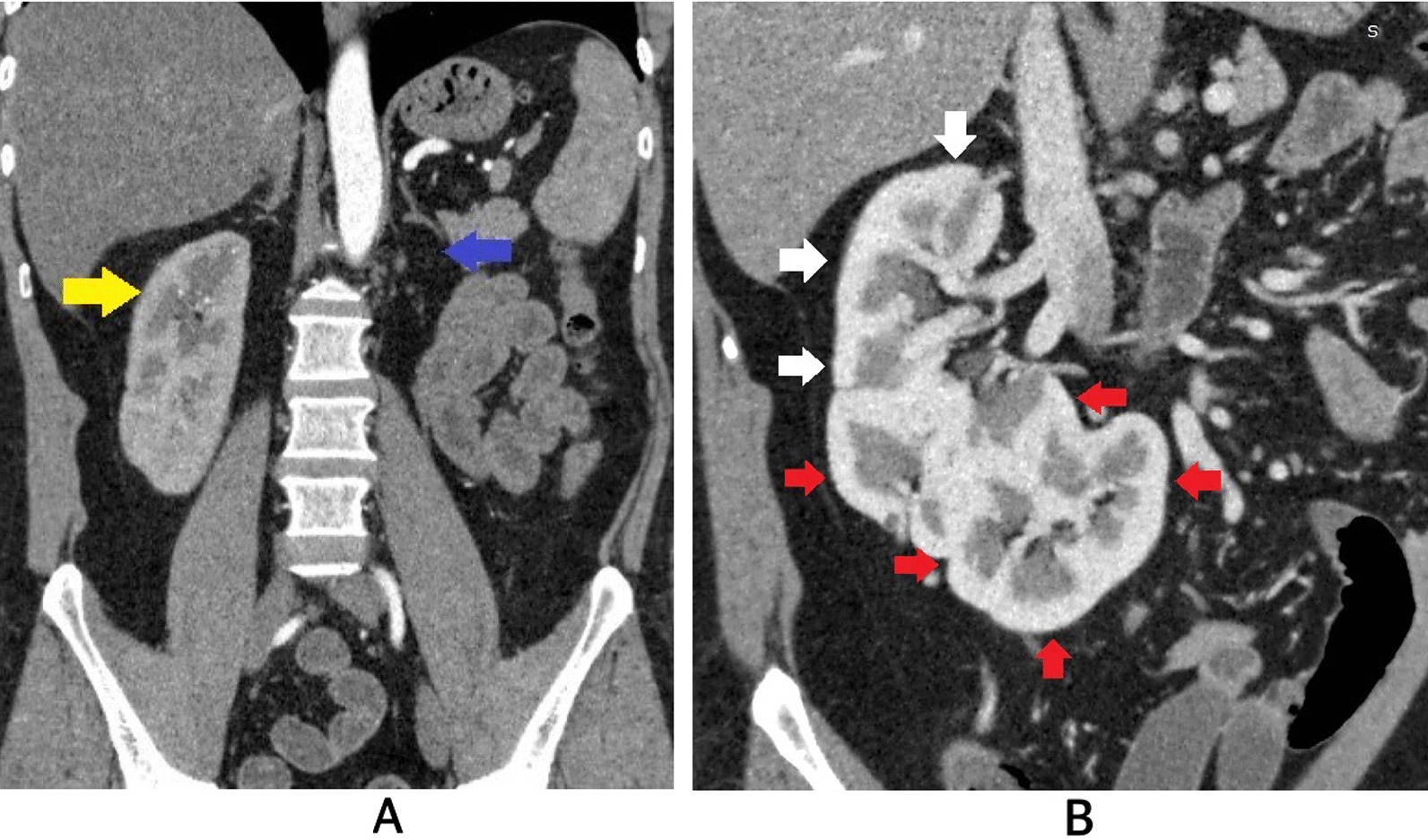
Contrast-enhanced computed tomography images in a soft tissue window. In **a**, a coronal section image at the level of bilateral renal fossa reveals the absence of the left kidney (purple arrow) and the presence of the right kidney (yellow arrow). **b** Displays an oblique reformatted image showing the right kidney formed by the fusion of two moieties (each shown by white and red arrows)

**Fig. 2:**
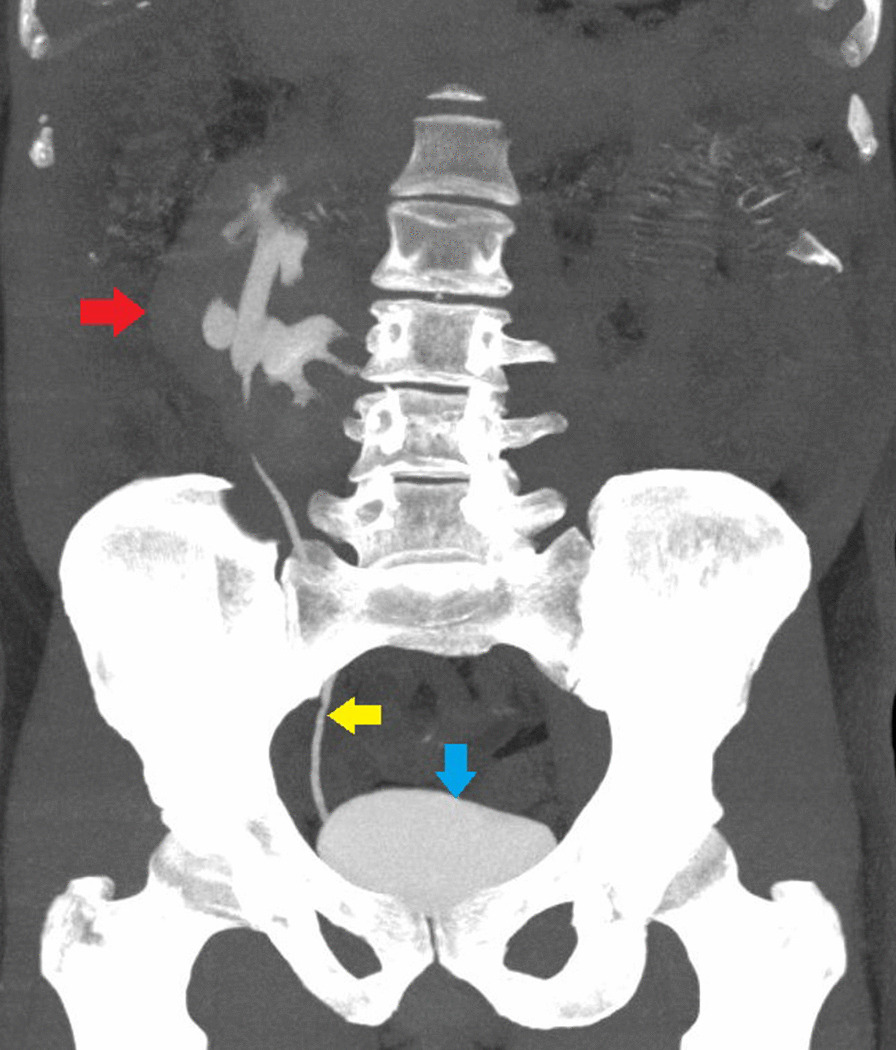
Contrast-enhanced computed tomography coronal section delayed phase images with maximum intensity projection depicting normal contrast opacification of the calyces, pelvis, and ureter of the crossed fused renal ectopia right kidney (red arrow). Some of the calyces and pelvis are malrotated, with only one ureter present (yellow arrow). The urinary bladder is indicated by the blue arrow

**Fig. 3:**
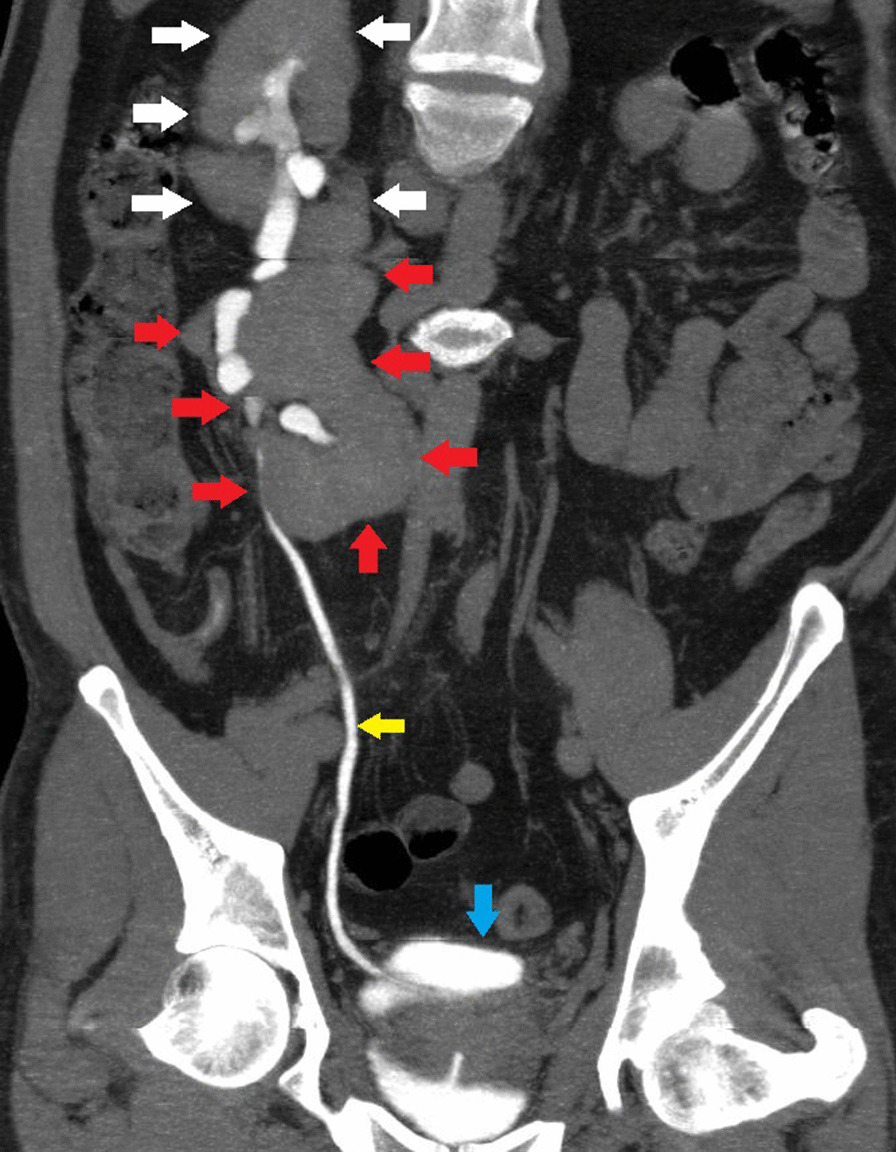
Contrast-enhanced computed tomography coronal section delayed phase curved reformatted images reveal two moieties in the right kidney (shown by white and red arrows), with only one ureter present (yellow arrow). The urinary bladder is shown by the blue arrow. The left renal fossa and other potential ectopic sites show the absence of the kidney

**Fig. 4:**
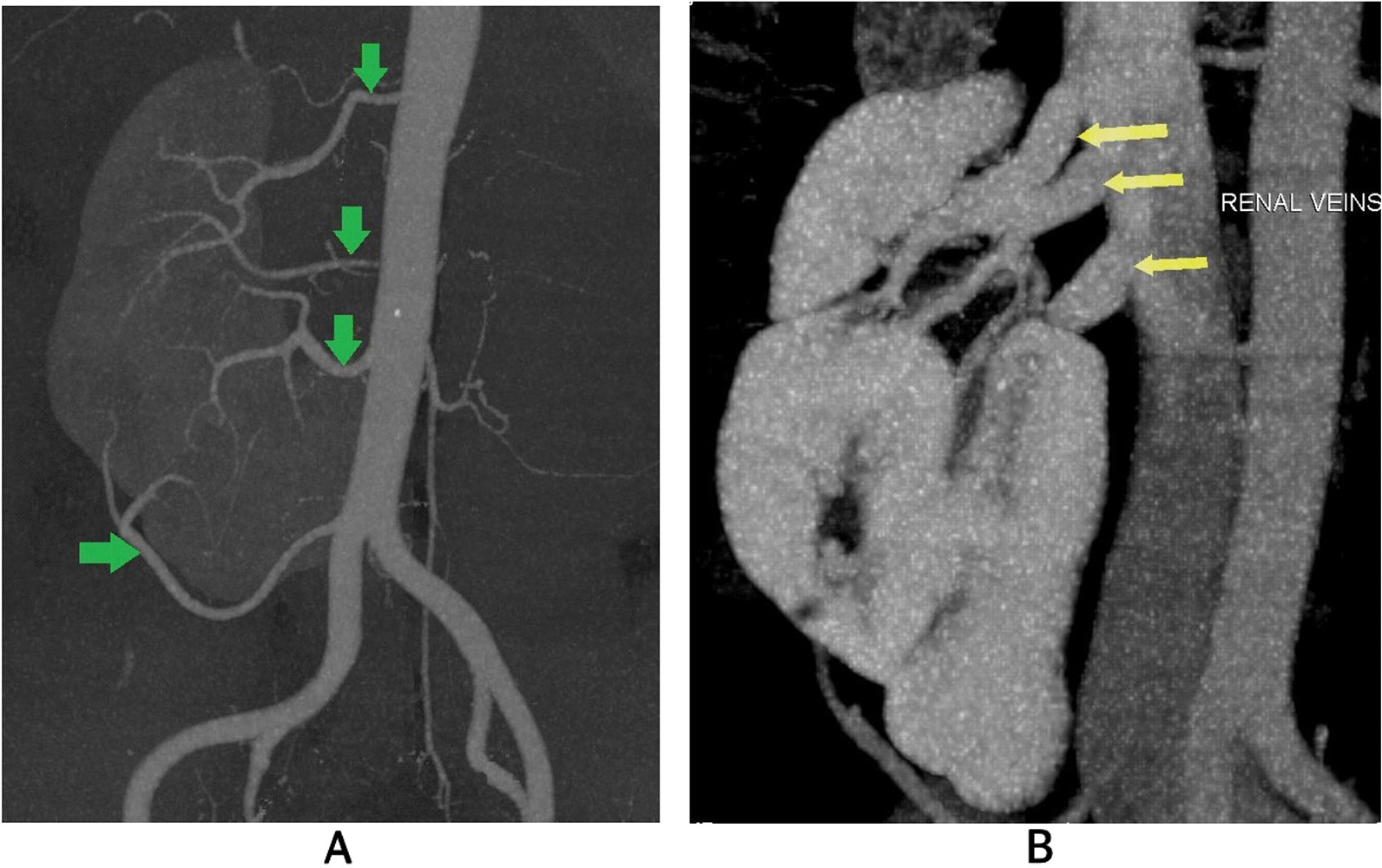
Contrast-enhanced computed tomography coronal section images illustrate the vascular anatomy of the right kidney in a CFRE. **a** exhibits four arteries supplying the right kidney (green arrows), while **b** shows three veins draining the kidney (yellow arrows) into the inferior vena cava

**Fig. 5:**
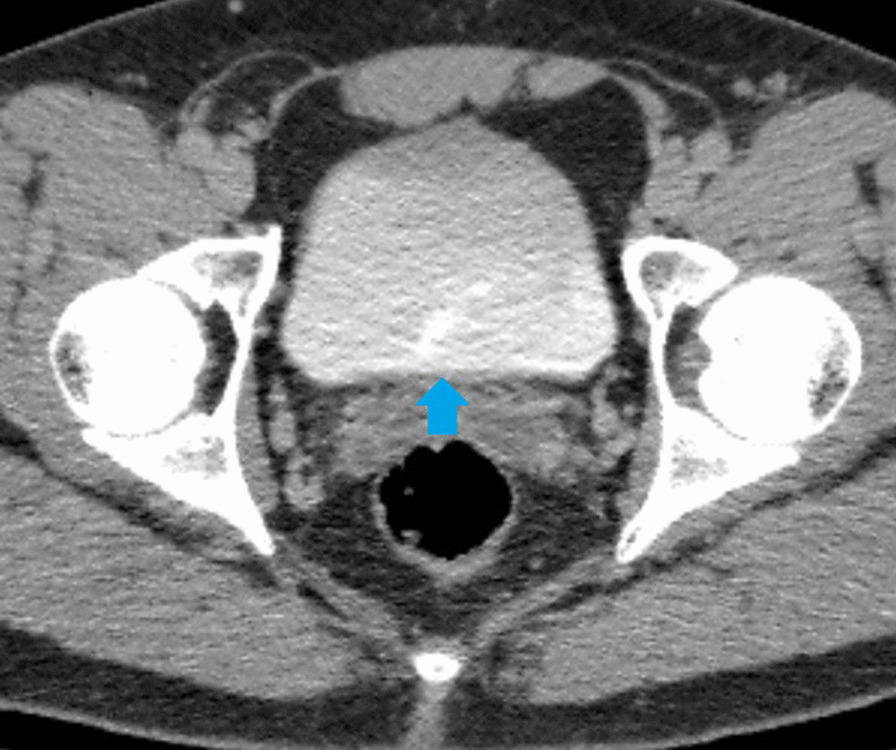
A contrast-enhanced computed tomography axial section delayed phase image at the level of the urinary bladder demonstrates normal opacification of the bladder (blue arrow)

Ultrasonography revealed the absence of the left kidney in the left renal fossa, with a malrotated, relatively large kidney observed on the right side. The possibility of crossed fused renal ectopia (CFRE) was suggested, leading to a recommendation for computed tomography (CT) urography. The CT urography confirmed right-sided CFRE (Fig. 1b) with a single ipsilateral ureter (Figs. 2, 3) with the left kidney located on the right side of the abdomen (Fig. 1a, b). The lower pole of the right kidney was fused with the upper pole of the left kidney, forming an “L”-type configuration (Fig. 1b). The renal pelvises of both kidneys were fused, and a single ureter on the ipsilateral side drained into the urinary bladder (Figs. 2, 3). Four renal arteries supplied the kidneys (Fig. 4a), and three renal veins drained them (Fig. 4b). The urinary bladder was essentially normal (Fig. 5). No calculi were present in the kidneys, ureter, or urinary bladder, and no other associated congenital anomalies were found. Echocardiography was performed to assess for cardiac anomalies, which showed normal results. Since the patient’s renal function remained intact and there were no issues stemming from the anomaly, he was counseled about the condition and advised to schedule regular follow-up appointments. The patient came to our outpatient department for a single follow-up visit after 6 months from the initial visit until the time we submitted this manuscript. He had no significant problems and exhibited good renal function.

## Discussion

Crossed fused renal ectopia (CFRE) ranks as the second most common renal fusion anomaly following horseshoe kidney, with an incidence ranging from 1 in 1300 to 1 in 7500 [3]. CFRE is characterized by the partial or complete fusion of both kidneys on the same side of the body. The occurrence of CFRE drained by a single ipsilateral ureter is a rare phenomenon, with only a few reported cases documented in the literature. McDonald and McClellan have identified six variations of CFRE [1, 2] (Table 1).

**Table 1:** McDonald and McClellan classification of CFRE

Category	Type of kidney	Description
Type 1	Inferior crossed fused ectopia	The upper pole of the ectopic kidney merges with the lower pole of the normal kidney, with both kidneys oriented cranio-caudally.
Type 2	Sigmoid or “S” shaped kidney	The hilum of the ectopic kidney faces laterally, while that of the normal kidney faces medially, resulting in an S-shaped mass after fusion.
Type 3	Unilateral lump kidney	There is fusion of two kidneys over a broad area, and the ureter from the ectopic kidney crosses the midline.
Type 4	“L”-shaped/tandem kidney	The ectopic kidney is positioned horizontally and fuses with the lower pole of the normal kidney.
Type 5	Unilateral disc kidney	Extensive fusion of two kidneys forming a disc-shaped mass.
Type 6	Superior crossed fused ectopia	The ectopic kidney is situated above the normal kidney and fuses with its upper pole.

The development of CFRE is intricately linked to the embryogenesis of the kidneys. Kidneys originate from the metanephros (of mesodermal origin) and the ureteric bud (derived from the Wolffian duct) [5]. While the embryogenesis of CFRE remains incompletely understood, two theories have been proposed: the ureteric theory and the mechanical theory [2]. According to the ureteric theory, excessive bending of the ureter and embryonal caudal end rotation of the metanephros prevent the ureter from meeting the metanephros on the same side, instead stimulating the metanephros of the contralateral side, resulting in CFRE. The mechanical theory suggests that an abnormally positioned umbilical artery obstructs the ascent of the developed kidney into its respective renal fossa. Consequently, the kidney follows the path of least resistance, leading to its placement on the contralateral side and the formation of CFRE.

However, the occurrence of CFRE drained by a single ureter cannot be fully explained by either of these theories, necessitating a reexamination of their premises. The arteries and veins associated with CFRE exhibit a wide range of variations [6]. CFRE is more commonly observed in males and typically affects the right side [7].

Vesicoureteral reflux (VUR) stands out as the most prevalent urological abnormality that commonly coexists with CFRE [4]. Alongside VUR, CFRE may be accompanied by other urogenital abnormalities such as pelviureteric junction obstruction (PUJO), hypospadias, undescended testis, and multicystic dysplastic kidney. Furthermore, CFRE may also present with non-urological abnormalities including tetralogy of Fallot, sacral agenesis, vaginal agenesis, and anal abnormalities.

The majority of CFRE cases are asymptomatic and often go undetected. However, some cases are incidentally discovered during imaging studies. CFRE can lead to urine stasis, fostering the development of renal calculi, infections, malignancies, and other complications [8]. In instances where CFRE is associated with VUR and PUJO, it may manifest as hydronephrosis [9]. Asymptomatic cases generally require no immediate intervention but necessitate regular follow-up to monitor renal function, assess for calculi formation, infections, and malignancies [10]. Surgical management is warranted for cases associated with VUR and PUJO. Treatment becomes imperative if calculi, infection, or malignancy are present at the time of diagnosis.

The biggest strength of this case report lies in its detailed documentation of a highly rare anomaly, accompanied by clear pictures. Few documentations exist to date describing this anomaly, making this manuscript a valuable reference for medical students, residents, and practitioners managing similar cases. However, our study has limitations. It focuses solely on a single case, necessitating further exploration of this anomaly’s characteristics and significance. Additional cases and longer-term follow-up studies are needed to deepen our understanding. Our follow-up period was limited to 6 months.

## Conclusion

The presence of a single draining ureter in CFRE signifies a rare renal anomaly, prompting inquiries into ureteric and mechanical theories. CFRE exhibits diverse variations in arteries, veins, and the ureter, emphasizing the necessity of thorough comprehension before embarking on any urological surgical interventions. CFRE engenders urinary stasis, thereby promoting the formation of calculi and infection. Consequently, diligent follow-up is crucial for monitoring renal function and averting the onset of calculi and infection.

## Data Availability

The data and material are not available in public repository, since this is a case report.
